# Immunogenicity of New Primary Immunization Schedules With Inactivated Poliovirus Vaccine and Bivalent Oral Polio Vaccine for the Polio Endgame: A Review

**DOI:** 10.1093/cid/ciy633

**Published:** 2018-10-30

**Authors:** Ananda S Bandyopadhyay, John F Modlin, Jay Wenger, Chris Gast

**Affiliations:** 1 Bill & Melinda Gates Foundation; 2 Biostatistics Consultant, Seattle, Washington

**Keywords:** inactivated poliovirus vaccine, bivalent oral poliovirus vaccine, monovalent oral poliovirus vaccine, polio eradication

## Abstract

In May 2016, countries using oral polio vaccine for routine immunization switched from trivalent oral poliovirus vaccine (tOPV) to bivalent type 1 and 3 OPV (bOPV). This was done in order to reduce risks from type 2 vaccine-derived polioviruses (VDPV2) and vaccine-associated paralytic poliomyelitis (VAPP) and to introduce ≥1 dose of inactivated poliovirus vaccine (IPV) to mitigate post-switch loss of type 2 immunity. We conducted a literature review of studies that assessed humoral and intestinal immunogenicity induced by the newly recommended schedules. Differences in seroconversion rates were closely associated with both timing of first IPV administration and number of doses administered. All studies demonstrated high levels of immunity for types 1 and 3 regardless of immunization schedule. When administered late in the primary series, a second dose of IPV closed the humoral immunity gap against polio type 2 associated with a single dose. IPV doses and administration schedules appear to have limited impact on type 2 excretion following challenge.

The Global Polio Eradication Initiative is close to interrupting wild polio virus (WPV) transmission worldwide, with only 22 cases of type-1 WPV reported in 2017 [1]. To achieve the goal of complete eradication of polio, it will be necessary to discontinue the use of all live, attenuated oral poliovirus vaccine (OPV) viruses that, in settings with low population immunity, have the potential to revert to neurovirulent circulating vaccine derived polioviruses (cVDPV). As the last naturally occurring WPV2 was isolated in 1999 and approximately 90 of all cVDPV outbreaks since 2000 have been attributed to type 2 viruses [2], a globally coordinated “switch” from trivalent OPV (tOPV) to bivalent type 1 and 3 OPV (bOPV) occurred in April 2016–May 2016, resulting in cessation of all use of OPV2 for routine childhood immunization worldwide [3]. To mitigate ongoing risk from type 2 polioviruses, the World Health Organization (WHO) Strategic Advisory Group of Experts (SAGE) recommended including 1 or more inactivated poliovirus vaccine (IPV) doses in the new routine infant immunization schedules [4]. However, an ongoing shortage of stand-alone IPV formulations since 2014 means that at least 44 countries have been prevented from introducing IPV or have suspended IPV use due to supply disruptions at different time points during and following OPV2 cessation [5]. In response, SAGE has encouraged the use of fractional doses delivered intradermally to extend supplies.

We performed a comprehensive review of randomized, controlled clinical trials (RCTs) to assess the immunogenicity of mixed and sequential IPV and bOPV schedules that are now in practice following the rapid transition in global polio immunization policies. This review summarizes key data from recent studies, focusing on the impact of such schedules on type 2 immunity, to inform global polio immunization policy until all use of OPV can be discontinued.

## METHODS

Eight RCTs published in 9 papers between November 2015 and March 2018 are included in this review, including 1 study in 4 Latin American countries reported in 2 papers (Table 1). These study designs reported mixed (IPV and bOPV given concomitantly at 1 or more time points in the primary series) or sequential (IPV and bOPV given sequentially but never concomitantly in the primary series) regimens for primary immunization. Studies were previously known to the authors or found by searching the PubMed database using search terms related to bOPV and IPV/fractional IPV (fIPV) regimens. Data from IPV-only and bOPV-only comparison arms were excluded.

**Table 1.** The Eight Identified Studies With the Groups Receiving Bivalent Type 1 and 3 Oral Poliovirus Vaccine and Inactivated Poliovirus Vaccine (IPV) (Full Dose [IPV]/fractional Dose [fIPV]) in This Review

First Author [Ref]	Year Study Was Initiated	Country(ies)	N^a^	Regimens Evaluated	Serology Time Points for Last Dose	OPV Challenge	Shedding Time Points, Post-challenge
Anand et al [6]	2012	Bangladesh	216	fIPV-bOPV-fIPV	18	tOPV(18)	19
O’Ryan et al [7]	2013	Chile	190	IPV(8)-bOPV(16)-bOPV(24)	28	mOPV2(28)	29, 30, 31, 32
			192	IPV(8)-IPV(16)-bOPV(24)	28	mOPV2(28)	29, 30, 31, 32
Sutter et al [8]	2013	India	180	bOPV(0)-bOPV-bOPV-bOPV/IPV	18	tOPV(18)	19, 22
			180	bOPV(0)-bOPV-bOPV-bOPV/IPV-IPV(18)	19	NA	NA
Sáez-Llorens et al [9]	2014	Panama	116	bOPV-bOPV-bOPV/IPV	18	mOPV2(18)	19, 20, 21
			117	bOPV-bOPV-bOPV/mIPV2HD^b^	18	mOPV2(18)	19, 20, 21
Asturias et al [10] Lopez-Medina et al [11]	2013	Colombia, Dominican Republic, Guatemala, Panama	310	bOPV-bOPV-bOPV/IPV	18	mOPV2(18)	19, 20, 21, 22
			590	bOPV-bOPV-bOPV/IPV-IPV(36)	40	mOPV2(40)	19, 20, 21, 22
Qiu et al [12]	2015	China	100	IPV(10)-bOPV(14–16)-bOPV(18–22)	30 days post-3rd dose	NA	NA
			100	IPV(10)-IPV(14–16)-bOPV(18–22)	30 days post-3rd dose	NA	NA
Taniuchi et al [13]	2015	Bangladesh	96	bOPV-bOPV-bOPV/IPV	NA	mOPV2(19–44)	1 week post-challenge
			103	bOPV-bOPV-bOPV/IPV-IPV(18)	NA	mOPV2(19–45)	1 week post-challenge
Saleem et al [14]	2014	Pakistan	360	bOPV(0)-bOPV-bOPV-bOPV/IPV	22	tOPV(22)	23

Regimens are given at weeks 6, 10, and 14, unless otherwise noted parenthetically. All time points are weeks. A slash (/) indicates more than 1 vaccine given at a particular time point. Sequence of the studies in the table is based on timing of publication.

Abbreviations: bOPV, bivalent type 1 and 3 OPV; fIPV, fractional-dose IPV; IPV, inactivated poliovirus vaccine; mOPV, monovalent type 2 OPV; mIPV2HD, monovalent type 2 high-dose IPV; NA, not applicable (data not reported in the articles); OPV, oral poliovirus vaccine; tOPV, trivalent oral poliovirus vaccine.

^a^Number randomized in each group.

In these studies, seroconversion was most frequently defined as a change from seronegative to seropositive (neutralizing antibody [NAb] titers ≥8) or, among seropositive patients, as NAb titers 4-fold or greater above the expected level accounting for decay in maternal antibodies [7–12, 14]. Most testing for poliovirus and polio antibodies was performed at the Centers for Disease Control and Prevention [7, 9–11, 14] or a specialized global polio eradication network laboratory in India using similar protocols [8]. For the study conducted in China, samples were tested at the National Institutes for Food and Drug Control in China [12]; for 1 of the Bangladesh studies, samples were processed at the International Centre for Diarrhoeal Disease Research, Bangladesh [13].

Seven of these studies evaluated proportions of vaccinees shedding poliovirus in stool 7 or more days after OPV challenge using quantitative reverse transcriptase polymerase chain reaction. Three evaluated proportion of shedders and concentrations of shed virus (log_10_ CCID_50_/g) through day 21 or day 28 post-challenge and computed a shedding index endpoint (SIE) to describe the extent of viral shedding as a single value from the average of the titers of shed virus in stool samples (log_10_ CCID_50_/g) collected on days 7, 14, 21, and/or 28 post-monovalent OPV2 (mOPV2) challenge [7–10]. In this way, the SIE captures both the magnitude and duration of viral shedding.

## RESULTS

### Humoral Immunity

Three studies [8–11] incorporated at least 3-dose bOPV regimens at birth and/or 6, 10, and 14 weeks with IPV coadministered with the third or fourth bOPV dose. Type 2 seroconversion rates were 69.3 [8], 74.8 [9], and 79.3 [11] (Table 2), producing a combined estimate of 75.4. In these studies, seroconversion rates for types 1 and 3 were uniformly high (>90%; Table 2). In the Pakistan study, 8 weeks after a fourth bOPV dose and 1 IPV dose, 94.9% and 96.0% of children seroconverted to types 1 and 3, respectively [14]. Studies in Chile (infants aged 8 weeks at first dose) and China (infants aged approximately 10 weeks at first dose) with IPV alone as the first dose followed by 2 bOPV doses also achieved high type 1 and 3 seroconversion rates (>98%), but only 77.4% and 55.8% type 2 seroconversion, respectively. In contrast, monovalent type 2 high-dose IPV (mIPV2HD) in Panamanian infants [9] achieved a type 2 seroconversion rate of 93.0%, significantly higher than conventional IPV (74.8%, *P* < .0001), with an estimated seroconversion rate closer to the bOPV + 2 IPV regimens from other studies. The Pakistan study was an investigation of different bOPV and IPV vaccination schedules compared with tOPV given to malnourished and normal infants at birth and 6, 10, and 14 weeks [14]. In normal infants assessed at age 14 weeks, after having received only bOPV up to that point, 17.3% (73/421) had already seroconverted to type 2. Of these, 277 then received IPV coadministered with a fourth bOPV dose; 8 weeks later at age 22 weeks, 51.3% (142/277) had seroconverted to type 2. In those children who had not already seroconverted to type 2 by 14 weeks, 44.3% (102/230) did so 8 weeks after 1 dose of IPV. In the 144 infants who only received a fourth bOPV dose without IPV, the seroconversion rate only increased from 18.1% (26/144) at 14 weeks to 19.4% (28/144) by 22 weeks. In control groups that received 4 doses of either IPV or tOPV, respective type 2 seroconversion rates were 84.1% (116/138) and 93.3% (125/134).

**Table 2.** Type 2 Seroconversion and Priming Where Available From 1, 2, and 3 Full or Fractional Doses of Inactivated Poliovirus Vaccine With or Without Bivalent Oral Poliovirus Vaccine

Author [Ref] Country(ies)	Schedule	Seroconversion Rate, n/N (%)					
		Type 2			Passive Type 2 Exposure (Seroconversion Before IPV/ Control Groups)	Type 1	Type 3
		4 (8^a^) Weeks After Final Vaccination	Priming: 1 Week After Challenge	Total (1 Week Post-challenge)		4 Weeks (8^a^) After Final Vaccination	
Anand et al [6] Bangladesh	fIPV-bOPV-fIPV	172/211 (81.5)	NA	NA	28/200 (14.0)^b^	202/211 (95.7)	198/211 (93.8)
O’Ryan et al [7] Chile	IPV(8)-bOPV(16)-bOPV(24)	130/168 (77.4)	19/29 (65.5)	142/15 (90.4)	NA	166/168 (98.8)	163/166 (98.2)
	IPV(8)-IPV(16)-bOPV(24)	169/176 (96.0)	NA	165/169 (97.6)	NA	178/178 (100)	177/177 (100)
Sutter et al [8] India	bOPV(0)-bOPV-bOPV-bOPV/IPV	115/166 (69.3)	NAd	150/156 (96.2)^d^	31/164 (18.9)^b^	165/166 (99.4)	165/166 (99.4)
	bOPV(0)-bOPV-bOPV-bOPV/IPV-IPV	155/155 (100)^c^	NA	NA	NA	155/155 (100)^c^	155/155 (100)^c^
Sáez-Llorens et al [9] Panama	bOPV-bOPV-bOPV/IPV	86/115 (74.8)	7/8 (87.5)	104/11 (91.2)	NA	105/115 (91.3)	113/115 (98.3)
	bOPV-bOPV-bOPV/mIPV2HD	107/115 (93.0)	1/2 (50)	110/112 (98.2)	NA	110/115 (95.6)	113/115 (98.2)
Asturias et al [10] Lopez-Medina et al [11] Colombia, Dominican Republic, Guatemala, Panama	bOPV-bOPV-bOPV/IPV	226/285 (79.3)	20/38 (52.6)^e^	260/283 (91.9)	19/198 (9.6)^b^	285/285 (100)	284/285 (99.6)
	bOPV-bOPV-bOPV/IPV-IPV(36)	534/535 (99.8)	NA	NA	21/189 (11.1)^f^	534/535 (99.8)	534/535 (99.8)
Qiu et al [12] China	IPV(10)-bOPV(14–16)-bOPV(18–22)	48/86 (55.8)	NA	NA	NA	85/86 (98.8)	85/86 (98.8)
	IPV(10)-IPV(14–16)-bOPV(18–22)	71/86 (82.6)	NA	NA	NA	81/86 (94.2)	85/86 (97.7)
Saleem et al [14] Pakistan	bOPV(0)-bOPV-bOPV-bOPV/IPV	142/277 (51.3)	NA	NA	28/144 (19.4)	263/277 (94.9)	266/277 (96.0)

For details of schedules, see Table 1. bOPV/IPV indicates concomitant administration of both vaccines at same visit.

Abbreviations: bOPV, bivalent type 1 and 3 OPV; fIPV, fractional-dose IPV; IPV, inactivated poliovirus vaccine; mOPV, monovalent type 2 OPV; mIPV2HD, monovalent type 2 high-dose IPV; NA, not applicable (data not reported in the articles); OPV, oral poliovirus vaccine; tOPV, trivalent oral poliovirus vaccine.

^a^Eight weeks after final vaccination in Pakistan study, Saleem et al [14].

^b^bOPV-only control group, 18 weeks.

^c^Evaluated 1 week after second IPV dose.

^d^Evaluated 4 weeks after tOPV challenge.

^e^Week 14, prior to IPV administration and after 2 bOPV doses. Only evaluated out of 210 randomized to 1 manufacturer group [5]

^f^ bOPV-only control group, 40 weeks.

Among individuals vaccinated with bOPV + 1 IPV and challenged with mOPV2 4 weeks following the final vaccination, more than half of the patients who had not seroconverted did so by 7 days after the challenge, indicating they were primed. Priming rates were 65.5%, 87.5%, and 52.6% (studies 7, 9, and 10, respectively; Table 2). In the study in India [8], priming in the bOPV + one IPV group was not assessed 1 week post-challenge although following a tOPV challenge, 86.4% of subjects who had not seroconverted to type 2 before challenge did so 4 weeks later. The studies in Chile [7], India [8], and 4 Latin America countries [10, 11] included groups that received bOPV + 2 IPV doses, each of which achieved seroconversion of ≥96% for type 2 and >99% for types 1 and 3 (Table 2). The study in China [12] with patients given 2 IPV doses followed by a single bOPV achieved seroconversion rates of 94.2%, 82.6%, and 97.7% for types 1, 2, and 3, respectively.

A sequential schedule of 2 fractional IPV doses with 1 bOPV dose [6] produced a type 2 seroconversion rate (81.5%) similar to or somewhat higher than the bOPV + 1 IPV dose regimens in the multicountry Latin American (79.3%) and Indian (69.3%) studies.

### Intestinal Immunity

After 1 IPV dose in addition to 2 to 4 bOPV doses, and following challenge with either mOPV2 or tOPV, day 7 post-challenge type 2 viral shedding rates were 80.5% (Chile [7]), 60.3% (India [8]), 78.3% (Panama [9]), and 74.6% (multicountry Latin America [11]); Table 3), with the 32.9% in Pakistan [14] being a notable outlier. The Pakistan [14] and India [8] studies demonstrated the lowest bOPV + 1 IPV seroconversion rates and the highest passive type 2 exposure rates prior to challenge as measured by the post-final vaccination seroconversion rate in the control groups given bOPV only (19.4% [measured at 22 weeks, following 4 bOPV doses] and 18.9% [measured at 18 weeks, following 4 bOPV doses], respectively). This comparison is complicated by the timing of assessment and potentially by the level of maternally derived antibodies. Both Pakistan and India studies had similar type 2 seroprotection levels at birth (83% and 85%, respectively), while the Chile and multicountry Latin American studies [7, 10] had pre-vaccination seroprotection rates of 64.7% and 47.6% at age 8 weeks and 6 weeks, respectively. The lower shedding rate among participants receiving 1 IPV dose in India compared with multicountry Latin American and Chilean studies persisted 28 days post-challenge (20.5% [India] vs 33.8% [multicountry Latin America] and 45.9% [Chile]), but no day 28 post-challenge data were available from the Pakistan study (Table 3). These comparisons are confounded by use of tOPV challenge (India and Pakistan studies) vs mOPV2 challenge (multicountry Latin American and Chilean studies). Notably, in the Chile study, the 1 IPV + 2 bOPV group had a statistically significantly lower SIE than the 2 IPV + 1 bOPV group.

**Table 3.** Type 2 Viral Excretion After Oral Challenge

Author [Ref] Country(ies)	Schedule	Proportion Shedding Type 2 Poliovirus, n/N (%)				Median 28-Day Shedding Index Endpoint (95% Confidence Interval)	Passive Type 2 Exposure (Day 0 Shedding)
		Day 7	Day 14	Day 21	Day 28		
Anand et al [6] Bangladesh	fIPV-bOPV-fIPV	122/211 (57.8)	NA	NA	NA	NA	NA
O’Ryan et al [7] Chile	IPV(8)-bOPV(16)-bOPV(24)	132/164 (80.5)	110/165 (66.7)	103/169 (60.9)	79/172 (45.9)	3.5 (2.8, 4.2)	5/173 (2.9)
	IPV(8)-IPV(16)-bOPV(24)	139/179 (77.7)	122/179 (68.2)	94/178 (52.8)	76/181 (42.0)	3.1 (2.6, 3.8)	2/181 (1.1)
Sutter et al [8] India	bOPV(0)-bOPV-bOPV/IPV	94/156 (60.3)	NA	NA	32/156 (20.5)	NA	10/156 (6.4)
Sáez-Llorens et al [9] Panama	bOPV-bOPV-bOPV/IPV	83/106 (78.3)	64/105 (60.9)	46/100 (46.0)	NA	4.1 (3.2, 4.8)^a^	NA
	bOPV-bOPV-bOPV/mIPV2HD	88/108 (81.5)	67/108 (62.0)	46/101 (45.5)	NA	4.0 (3.2, 4.9) ^a^	NA
Asturias et al [10] Lopez-Medina et al [11] Colombia, Dominican Republic, Guatemala, Panama	bOPV-bOPV-bOPV/IPV	211/283 (74.6)	162/280 (57.9)	126/281 (44.8)	95/281 (33.8)	2.5 (2.3, 2.9)	3/79 (3.8)^b^
	bOPV-bOPV-bOPV/IPV-IPV(36)	367/528 (69.5)	273/524 (52.1)	153/523 (29.3)	129/529 (24.4)	2.2 (1.8, 2.3)	
Taniuchi et al [13] Bangladesh	bOPV-bOPV-bOPV/IPV	61/80 (76.3)	NA	NA	NA	NA	NA
	bOPV-bOPV-bOPV/IPV-IPV(18)	60/80 (75.0)	NA	NA	NA	NA	NA
Saleem et al [14] Pakistan	bOPV(0)-bOPV-bOPV-bOPV/IPV	23/70 (32.9)	NA	NA	NA	NA	NA

Abbreviations: b, bivalent type 1 and 3; f, ; IPV, inactivated poliovirus vaccine; m, ; mIPV2HD, monovalent type 2 high-dose IPV; NA, not applicable (data not reported in the articles); OPV, oral poliovirus vaccine; t, .

^a^Shedding index endpoint calculated only from days 7, 14, and 21 post-challenge.

^b^Subsample of parallel bOPV-only control groups [6].

In the Bangladesh study by Taniuchi et al [13] where vaccinees ranging in age from about 19 weeks to 45 weeks (mean, approximately 31 weeks) received bOPV and 1 or 2 conventional IPV doses, proportions of vaccinees shedding poliovirus in stool 1 week following mOPV2 challenge (76.3% and 75.0%, respectively) were similar to both the 1-IPV and 2-IPV groups challenged 4 weeks following the final vaccination in the multicountry Latin America, Chile, and Panama studies (Table 3).

Across and within studies, receipt of 2 IPV doses compared with 1 dose was associated with a marginal reduction in viral shedding, particularly beyond the first 2 weeks after challenge. Proportions of participants shedding virus on post-challenge days 21 and 28 were lower for those given a second IPV dose, and the total extent of shedding (measured by SIE) was also lower in these groups. In contrast, in the Chile study, patients who received 3 IPV doses and no bOPV displayed viral shedding that was significantly higher than in either bOPV + IPV arms [7].

Despite a substantially higher seroconversion rate following mIPV2HD compared with conventional IPV in the Panama study, the rate and extent of viral shedding was not substantially different between the 2 regimens [9].

## DISCUSSION

This is the first summary of the evolving evidence base of new polio immunization schedules that have been in practice since the global cessation of trivalent OPV use in May 2016 [3]. Such schedules would continue until cessation of bOPV use, approximately 1 year following certification of eradication by the WHO Global Certification Commission, which could occur in the early 2020's. Throughout this transition phase, evolving risks of WPV1 transmission and continued vaccine-derived circulation will have to be addressed with effective vaccination options with IPV and bOPV, with VDPV2 outbreak response use of mOPV2 if necessary. The literature reviewed and summarized here specifically considers these new primary immunization schedules and assesses the impact on both humoral and intestinal immunogenicity.

With certification of WPV2 eradication, the primary focus of the global program is to ensure adequate type 1 and 3 immunity with bOPV and IPV. All regimens evaluated in these RCTs induce high type 1 and 3 seroconversion rates (>85% across different regimens) 4 weeks following the last dose in a schedule. This is reassuring, and such high levels of immunity should lead to interruption of wild polio transmission if adequate coverage can be maintained, supplemented by vaccination campaigns with bOPV in at-risk areas.

Humoral immunity against type 2 polioviruses, measured by seroconversion, is important for individual protection against paralytic poliomyelitis but is a weak indicator of intestinal immunity that has implications for person-to-person transmission of the virus [15]. Across the different dose regimens we reviewed, seroconversion rates to type 2 poliovirus varied primarily with age at first IPV administration. Studies evaluating a 6–10–14 week regimen of bOPV with first IPV dose at 14 weeks had a combined type 2 seroconversion rate of 75.4%. The humoral responses to 1 or 2 doses of IPV are higher than prior assessments, probably due to the late administration of IPV that minimizes the negative impact of maternally derived antibodies [16]. Lower seroconversion rates from the bOPV + 1 IPV group of Indian infants [8] compared with Latin American infants [10, 11] appear in tandem with higher passive type 2 exposure, evidenced in the respective bOPV-only control groups, providing a probable explanation for the difference. In Pakistan, a birth dose of bOPV followed by bOPV at 6–10–14 weeks and 1 dose of IPV at 14 weeks produced low type 2 seroconversion rates (51.3%), possibly due to high levels of maternal antibodies at baseline and widespread (19.4%) passive exposure to circulating type 2 vaccine viruses confounding the impact of IPV at 14 weeks. In contrast, the Chile and China studies evaluating sequential schedules [7, 12], where IPV was given at 8 or 10 weeks without concomitant bOPV, reported type 2 seroconversion rates of 77.4% and 55.8%, respectively. Studies that evaluated the 2-dose impact of IPV (India, Chile, Latin America) reported uniformly high seroconversion rates of ≥96% for type 2 (Table 2). The China study reported a lower type 2 seroconversion rate (82.6%) in a sequential IPV-bOPV schedule.

Priming is measured by a rapid (within 1 week) seroconversion to a homotypic vaccine administration among those who did not seroconvert with earlier dose(s). Across all studies, the seroconverted-or-primed rates (Table 2) elicited by mOPV2 or tOPV challenge were consistently higher than 90%, allowing only a marginal gain with a second IPV dose where applicable. More than half (60%, Latin America) and up to 88% (Panama) of patients receiving only 1 IPV with bOPV were primed for type 2. The total proportion of those either seroconverted or primed among all 1-IPV recipients was consistently >90%, indicating 1-dose IPV recipients may elicit rapid, high immune responses to mOPV2 in the rare event of an outbreak in the post-type 2 cessation era. However, the clinical and epidemiologic significance of priming and its correlation with protection from paralysis remain unclear.

The most widely accepted surrogate for describing varying levels of intestinal immunity is resistance from viral excretion following an oral challenge with the live attenuated vaccine [17]. In the past, several studies examined mixed or sequential IPV and tOPV regimens for primary immunization and reported humoral and intestinal immunity in such schedules [8, 18–20]. With new IPV–bOPV regimens and mOPV2 challenge following the last dose of study vaccine, the studies reviewed here provide a unique opportunity to evaluate primary intestinal immunogenicity against type 2 from 1 or more doses of IPV. Use of mOPV2 challenge in the study designs in infant bOPV and IPV immunization schedules is expected to closely simulate the endgame schedules and type 2 virus reintroduction situation. We found that, with the exception of the Pakistan study, the majority of challenged vaccinees shed type 2 virus in stool (60.3%–80.5%), consistent with prior experience in IPV-only vaccinated patients receiving an OPV challenge. The lower day 7 shedding (32.9%) reported in Pakistan may be explained by extensive prior type 2 exposure through passive circulation of Sabin 2 in the community. We noted a marginal reduction of shedding in recipients of 2 IPV doses, particularly beyond the first 2 weeks following challenge, with both the proportion of participants shedding virus on post-challenge days 21 and 28 and a lower overall magnitude of shedding compared with 1-dose IPV recipients. It is not clear if such a narrow impact would have any meaningful clinical or epidemiologic significance. Recently, the impact of IPV and bOPV on primary intestinal immunogenicity has also been assessed by measuring poliovirus-specific antibodies in the stool using samples from RCTs reported in this review [21, 22]. These novel assays confirm that there is limited impact of IPV, both conventional and high dose, on intestinal mucosal immune responses or in limiting viral shedding upon challenge. We cannot determine whether the lower extent of shedding seen in the multicountry Latin America study compared with the Chilean study represents a difference due to regimen or differences between the included countries, as these variables are confounded.

Studies evaluating immunogenicity of higher or lower than conventional IPV doses yielded interesting results. High-dose mIPV2HD, with 4-fold more type 2 antigen than conventional IPV, administered at 14 weeks concomitantly with a third bOPV dose produced a markedly higher seroconversion rate (93.0%) compared with conventional trivalent IPV (74.8%) [9]. In contrast, 2 intradermal doses of fIPV, with one fifth of the intramuscular dose, conferred type 2 protection comparable to 1 full dose of intramuscular IPV when given with bOPV. Induction of types 1 and 3 humoral immunity as measured by seroconversion from the fractional doses also appeared to be adequate. Interestingly, neither fIPV nor mIPV2HD seemed to have any meaningful impact in reducing virus excretion compared with the full-dose intramuscular option (Table 3). This finding emphasizes that irrespective of the dose and route of administration, IPV has limited impact on primary intestinal immunity. However, as noted in studies designed to evaluate the boosting impact of IPV on OPV-vaccinated children, an additional dose of IPV in OPV-exposed children may have an equivalent or better impact in reducing shedding compared with a dose of OPV, and such boosting of intestinal immunity probably lasts for at least 1 year [23–25].

This review has limitations. Inclusion is restricted to primary infant routine immunization schedules with both bOPV and IPV. Differences in analytic methodologies of these RCTs in the absence of original clinical data from some studies could raise issues with generalization of the data used for interpretation. Similarly, age differences and timing of sampling could impact comparability of data across the studies. None of the reviewed studies included any assessment of oropharyngeal excretion of vaccine virus post-challenge, so our assessment of IPV impact on polio type 2 mucosal immunity is limited to intestinal immunity.

To summarize, new routine polio endgame immunization schedules of bOPV and 1 IPV dose provide high levels of individual protection against type 1 and 3 and moderate levels of protection against type 2. A second dose of IPV closes the humoral immunity gap for type 2 largely irrespective of the primary immunization schedule. With current supply and cost constraints with IPV, dose-sparing through 2 intradermal doses of fIPV could be an important option in the near term and the long term as the doses appear to provide type 2 humoral and intestinal immunity comparable with 1 full dose of intramuscular IPV [26]. Increasing the antigen content of IPV appears to substantially improve humoral immunity, as seen with mIPV2HD. However, the lack of any advantage for intestinal immunity and cost and manufacturing complexities limit the usefulness of such a vaccine candidate. mOPV2, the current outbreak response option in the OPV2 cessation era, elicited robust and prompt humoral responses in infants previously vaccinated with bOPV and IPV. Minor variances noted in intestinal mucosal immunity and priming in these schedules are intriguing and merit further research. The Chile study showed a reduction in the extent of viral shedding associated with additional bOPV doses rather than IPV, suggesting a role for intestinal cross-protection from bOPV, but the clinical and epidemiologic impacts, if any, from these observations remain unclear.

Interruption of WPV1 transmission should be within reach with the current vaccine options, as demonstrated by the excellent immunogenicity of IPV and bOPV primary series. Research and development efforts for improved, safer vaccine options and polio antiviral drugs may strengthen the likelihood of sustaining polio-free status for the long term by eliminating all risks of polioviruses [2]. Continued, intensified vaccination efforts through routine immunization and supplementary immunization activities will be critical in achieving the necessary levels of protection to enable long-term, permanent success of the eradication program.

## Notes

***Acknowledgments.*** The authors are grateful to all the patients and their parents for participating in the trials reviewed here. Thanks to the sponsors; principal investigators; laboratory partners; vaccine suppliers; national, regional, and global authorities; and other key partners who contributed to the planning and implementation of the studies reported here. The authors also thank Keith Veitch (keithveitch communications, Amsterdam, Netherlands) for help in the final phase of formatting and cleaning up the manuscript and Ricardo Rüttimann (Fighting Infectious Diseases in Emerging Countries) for facilitating this support.

***Financial support.*** No specific funding was received for this review article, and the authors are salaried employees or consultants with the Bill & Melinda Gates Foundation, which has no financial interests in the work.

***Supplement sponsorship.*** This article appears as part of the supplement “Polio endgame and beyond: Vaccine choices, transmission dynamics, and surveillance implications,” sponsored by The Bill & Melinda Gates Foundation.

***Potential conflicts of interest.*** All authors: No reported conflicts of interest. All authors have submitted the ICMJE Form for Disclosure of Potential Conflicts of Interest. Conflicts that the editors consider relevant to the content of the manuscript have been disclosed.
